# Levels of Complement Components in Children With Acute COVID-19 or Multisystem Inflammatory Syndrome

**DOI:** 10.1001/jamanetworkopen.2023.1713

**Published:** 2023-03-24

**Authors:** Anuradha Rajamanickam, Pavan Kumar Nathella, Aishwarya Venkataraman, Bindu Dasan, Sulochana Putlibai, Shaik Fayaz Ahamed, Nandhini Selvaraj, Kalaimaran Sadasivam, Balasubramanian Sundaram, Thomas B. Nutman, Subash Babu

**Affiliations:** 1 National Institutes of Health-National Institute for Research in Tuberculosis, International Center for Excellence in Research, Chennai, India; 2 Indian Council of Medical Research-National Institute for Research in Tuberculosis, Chennai, India; 3 Kanchi Kamakoti CHILDS Trust Hospital, Chennai, India; 4 Laboratory of Parasitic Diseases, National Institute of Allergy and Infectious Diseases, National Institutes of Health, Bethesda, Maryland

## Abstract

**Importance:**

Multisystem inflammatory syndrome in children (MIS-C) is a severe and unrestrained inflammatory response with multiorgan involvement, which occurs within a few weeks following the resolution of acute SARS-CoV-2 infection. The complement system is a vital part of the innate immune system and plays a role in COVID-19 pathogenesis.

**Objective:**

To examine and compare the levels of complement components and regulators along with complement activation products in the different clinical spectrum of children with SARS-CoV-2 and a control group.

**Design, Setting, and Participants:**

This cross-sectional study analyzed children with MIS-C admitted to a single hospital in India from June through September 2020. Eligible participants were children who were hospitalized of either sex, aged 1 to 18 years. Data were analyzed August 2022.

**Measures:**

Levels of complement components and regulators along with complement activation products in all the groups of children. Mann-Whitney *U* test and Kruskal-Wallis analysis were used to compare the complement component levels, and Spearman rank correlation analysis was used to describe the association between complement components and laboratory and biochemical parameters.

**Results:**

A total 145 children were included (median age, 5 years [range, 1 month-17 years); 84 [58%] male): 44 children with MIS-C, 33 with acute COVID-19 (reverse transcriptase–polymerase chain reaction [RT-PCR] positive), 47 with convalescent COVID-19 (immunoglobulin G–positive non-MIS-C) and 21 children for a control group (both serology and RT-PCR negative). Children with MIS-C and COVID-19 had higher levels of C1q (geometric mean [SD]: MIS-C, 61.5 [18.5] ng/mL; acute COVID-19, 56.9 [18.6] ng/mL; controls, 24.1 [3.3] ng/mL), C2 (MIS-C, 605.8 [219.7] ng/mL; acute COVID-19, 606.4 [167.7] ng/mL; controls, 255.9 [73.3] ng/mL), C3 (MIS-C, 318.2 [70.7] ng/mL; acute COVID-19, 237.7 [61.8] ng/mL; controls, 123.4 [15.7] ng/mL), C4b (MIS-C, 712.4 ng/mL; acute COVID-19, 640.7 ng/mL; controls, 351.5 ng/mL), C5 (MIS-C, 1487 ng/mL; acute COVID-19, 1364 ng/mL; controls, 561.9 ng/mL), C5a, (MIS-C, 2614.0 [336.2] ng/mL; acute COVID-19, 1826.0 [541.0] ng/mL; controls, 462.5 [132.4] ng/mL), C3b/iC3b (MIS-C, 3971.0 [635.1] ng/mL; acute COVID-19, 3702.0 [653.9] ng/mL; controls, 2039.0 [344.5] ng/mL), and factor B (MIS-C, 47.6 [7.8] ng/mL; acute COVID-19, 44.6 [6.3] ng/mL; controls, 27.5 [5.0] ng/mL), factor D (MIS-C, 44.0 [17.2] ng/mL; acute COVID-19, 33.8 [18.4] ng/mL; controls, 21.3 [6.1] ng/mL), and factor H (MIS-C, 53.1 [4.0] ng/mL; acute COVID-19, 50.8 [5.7] ng/mL; controls, 43.6 [3.8] ng/mL) in comparison with convalescent and control children. In addition, children with MIS-C had significantly elevated levels of C3 (318.2 [70.7] ng/mL vs 237.7 [61.8] ng/mL), C5a (2614 [336.2] ng/mL vs 1826 [541.0] ng/mL), and mannose-binding lectin (79.4 [12.4] ng/mL vs 69.6 [14.7] ng/mL) in comparison to children with acute COVID-19. Levels of some of these analytes at admission (ie, pretreatment) were more elevated in children with MIS-C who needed pediatric intensive care unit (PICU) support as compared with those who did not require PICU support, and in children with COVID-19 who developed moderate to severe disease compared with those who developed mild disease. Overall, MIS-C and acute COVID-19 were associated with the hyperactivation of complement components and complement regulators.

**Conclusions and Relevance:**

In this cross-sectional study, the complement system was associated with the pathogenesis of MIS-C and COVID-19 in children; complement inhibition could be further explored as a potential treatment option.

## Key Points

**Question:**

Are multisystem inflammatory syndrome in children (MIS-C) and COVID-19 in children associated with alterations in complement expression and function?

**Findings:**

In this cross-sectional study with 145 participants, MIS-C and COVID-19 were associated with higher levels of complement components, activation products, and regulators. Additionally, complement levels pretreatment were found to be potential biomarkers of disease severity in MIS-C and COVID-19.

**Meaning:**

The findings of this study suggest that complement activation was associated with the pathogenesis of MIS-C and acute COVID-19 and is a potential biomarker of disease severity.

## Teaser

This cross-sectional study of children treated in an Indian hospital examines associations of multisystem inflammatory syndrome and COVID-19 with alterations in complement expression and function.

## Introduction

Multisystem inflammatory syndrome in children (MIS-C) or pediatric inflammatory multisystem syndrome temporally associated with SARS-CoV-2 infection (PIMS-TS)^1,2^ are severe postinfectious hyperinflammatory sequela in children who are positive for current or recent SARS-CoV-2 infection by reverse transcriptase–polymerase chain reaction (RT-PCR), serology, or antigen test; or have exposure to a suspected or confirmed COVID-19 case within 4 weeks prior to the onset of symptoms. The clinical spectrum of MIS-C varies from mild to severe life-threatening illnesses with shock and multiorgan dysfunction (MODS), sometimes leading to death.^3,4,5,6^

The complement system plays a crucial role in host defense.^7,8^ The complement system is necessary for protection against viral infections, and its excessive activation and dysregulation can lead to systemic multiorgan injury.^9^ Recently, there have been reports that the dysregulation of complement proteins are involved in the COVID-19 disease pathology in adults and children.^8,10,11,12,13,14^ The immunological characteristics of MIS-C have been shown to include the activation of complement pathways and modification of innate and adaptive immune responses.^15,16^ The SARS-CoV-2 nucleocapsid protein has been shown to activate the lectin pathway,^8,17^ whereas the spike protein S activates the alternative pathway.^16^ Previous adult studies have demonstrated elevation of C5a and soluble C5b-9 in patients with COVID-19,^10,18,19^ as well as deposition of activated complement proteins in injured tissues and organs.^11,20^ Previous reports determined that MIS-C and acute COVID-19 children were associated with highly elevated levels of activation markers of the classical, alternative, and terminal pathways.^21,22^ Children with SARS-CoV-2 infection exhibited elevated levels of soluble C5b9, which correlated with disease severity.^13,22,23,24,25^ However, there remains a paucity of information on the degree and the possible involvement of complement in the pathogenesis of MIS-C and acute COVID-19 in children, especially from low middle-income countries. Therefore, we have performed an in-depth evaluation of the complement system in children with MIS-C, acute COVID-19, and convalescent COVID-19. In this cross-sectional study, we wanted to have a comprehensive view of the role of the complement system in the pathogenesis of MIS-C and COVID-19, and to determine if is there any association between the complement activation and children with MIS-C and COVID-19.

## Methods

The study was approved by the Kanchi Kamakoti CHILDS Trust Hospital (KKCTH)-CHILDS Trust Medical Research Foundation ethics committee and the National Institute for Research in Tuberculosis ethics committee. The study was also registered at Clinical Trials Registry India. Written informed consent or assent was obtained from all the children or their caretakers in this study. Children below age 12 years were included in the study after informed consent from the caretaker. The assent form was obtained from the children who were aged between 12 and 18 years. This study followed the Strengthening the Reporting of Observational Studies in Epidemiology (STROBE) reporting guideline for cross-sectional studies.

### Study Population and Procedures

The study population included children of either sex, aged 1 to 18 years, admitted to KKCTH in Chennai, India from June 1, 2020, to September 30, 2020. We included 21 children with a similar median age and sex, who were both SARS-CoV-2 RT-PCR negative and seronegative and presented to the hospital for elective procedures (controls). They had no other comorbid conditions and no history of COVID-19 contact.^26,27^ The clinical, demographic, and basic laboratory findings of these children have been previously described in detail (eTable 1 in Supplement 1).^26,28,29^ Children were categorized into 4 groups: COVID-19 (RT-PCR positive), MIS-C, seropositive (IgG positive non-MIS-C), and control (both serology and RT-PCR negative). Blood samples were collected from children prior to the initiation of any treatment. Plasma was isolated and used for measuring multiple parameters. COVID-19 disease and severity of COVID-19 were defined according to the Ministry of Health and Family Welfare guidelines^30^ issued by the Government of India and children with MIS-C or PIMS-TS were diagnosed according to the Royal College of Pediatrics and Child Health (RCPCH) case definition for PIMS-TS.^2^ Blood was collected into ethylenediaminetetraacetic acid tubes (BD Biosciences), plasma was obtained by centrifugation, and frozen at −80 °C until transferred to the National Institute for Research in Tuberculosis (NIRT) for serological and immunological analysis on ice. Peripheral blood sampling in all children was done prior to receiving any immunomodulatory treatment. Multiplex assays on all samples were performed at the same time to limit batch effect. Study staff involved in immunological and serological assays were masked to clinical data.

### SARS-CoV-2 Testing

SARS-CoV-2 real-time RT-PCR was performed by Indian Council of Medical Research (ICMR) approved laboratories. Serology was performed using SARS-CoV-2 IgG chemiluminescence antibody assay (YHLO Biotechnology Corporation) according to the manufacturer’s instructions. An IgG antibody titer of 10 AU/mL or above was considered positive.

### Measurement of Complement Cascade Proteins and Complement Regulatory Proteins

Systemic levels of C1q, C2, C3, C3b/iC3b, C4, C4b, C5, C5a, mannose-binding lectin (MBL) complement proteins and complement regulatory proteins like factor B, factor D, factor H, and factor I were determined using bead-based multiplex complement assay kits (Luminex Corporation). The lowest detection limits were as follows: C2, 1.4 ng/mL; C4b, 1.4 ng/mL; C5, 2.7 ng/mL; C5a, 4.1 ng/mL; complement factor D (adipson), 0.07 ng/mL; MBL, 0.1 ng/mL; complement factor I, 0.7 ng/mL; C1q, 0.1 ng/mL; C3, 0.3 ng/mL; C3b/iC3b, 8.2 ng/mL; C4, 0.6 ng/mL; complement factor B, 0.1 ng/mL; and complement factor H, 0.04 ng/mL.

### Statistical Analysis

For analysis, children were categorized into 4 groups: COVID-19 (RT-PCR positive), MIS-C, seropositive (IgG positive non-MIS-C), and control (both serology and RT-PCR negative). Geometric means (GM) were used for measurements of central tendency. Continuous variables are presented as medians and IQRs, and categorical variables are reported as numbers and proportions. Comparison between the groups was performed using the Mann-Whitney *U* test. Statistically significant differences between children in the MIS-C, COVID-19, seropositive, and control groups were analyzed using the Kruskal-Wallis test with Dunn multiple comparisons. A Mann-Whitney test was used to compare the levels of complement proteins between children with MIS-C admitted to pediatric intensive care unit (PICU) care and those who did not require PICU admission as well as between COVID-19 children with mild disease and those with moderate to severe disease. *P* ≤ .05 was considered statistically significant and all tests were 2-sided. Analyses were performed using Graph-Pad PRISM Version 9.0 (GraphPad Software). The data were analyzed using R version 4.2.1 within the RStudio platform (R Project for Statistical Computing).

## Results

### Baseline Characteristics

Our study included 145 children, as described previously,^26^ which included 33 children with COVID-19, 44 with MIS-C, 47 seropositive, and 21 control (eTable 1 in Supplement 1). The median age was 5 years (range, 1 month-17 years), and 84 children (58%) were male. All children with COVID-19 were SARS-CoV-2 RT-PCR positive and all children with MIS-C were seropositive (IgG). Of the 44 children with MIS-C, 23 children needed PICU admission, indicating severe disease, while 21 children were treated in the hospital wards, indicating mild or moderate disease. Of the 33 children with COVID-19, 22 (67%) presented with mild symptoms, 3 (9%) had moderate symptoms, 2 (6%) had severe symptoms needing PICU care, and 6 (18%) children were asymptomatic. Among the 47 children with seropositive non-MIS-C, 30 (64%) had no systemic symptoms and were admitted to the hospital for elective procedures. Children in the control group (21 participants) were both SARS-CoV-2 RT-PCR–negative and seronegative. They had similar median (IQR) age (6 [1-15] years) and sex (11 [52%] male), had no systemic symptoms, no other comorbid conditions, and no history of COVID-19 contact.

### Levels of Complement Activation Among Children With MIS-C and Acute COVID-19

**Figure 1.**
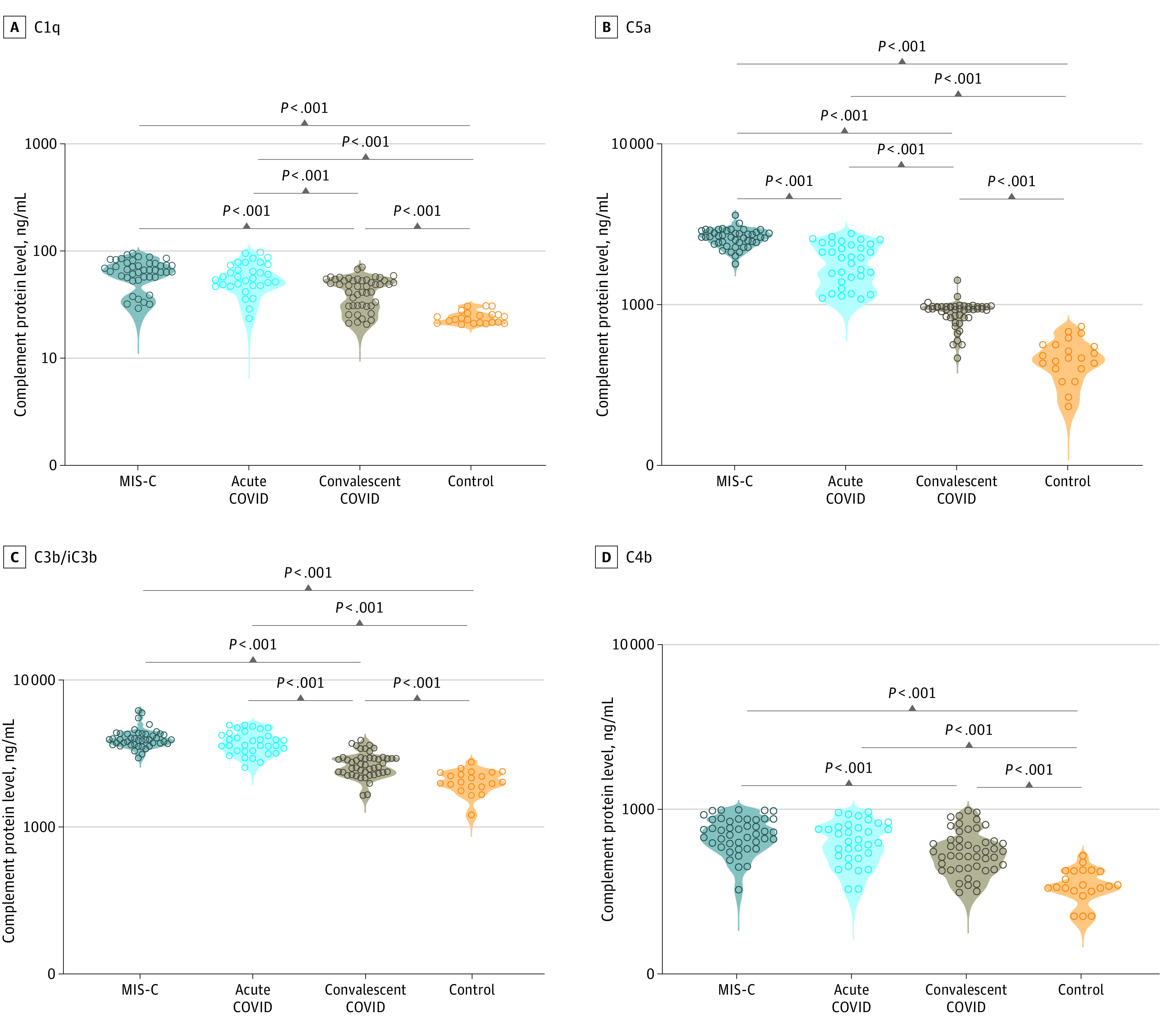
Complement Activation Products and Complement Components Activation in Children With Multisystem Inflammatory Syndrome in Children (MIS-C) and Acute COVID-19 Groups included plasma samples from 44 children with MIS-C, 33 with acute COVID-19, 47 with convalescent COVID-19, and 21 in the control group. Each circle represents a single individual. *P* values were calculated using the Kruskal-Wallis test with Dunn post hoc for multiple comparisons.

**Figure 2.**
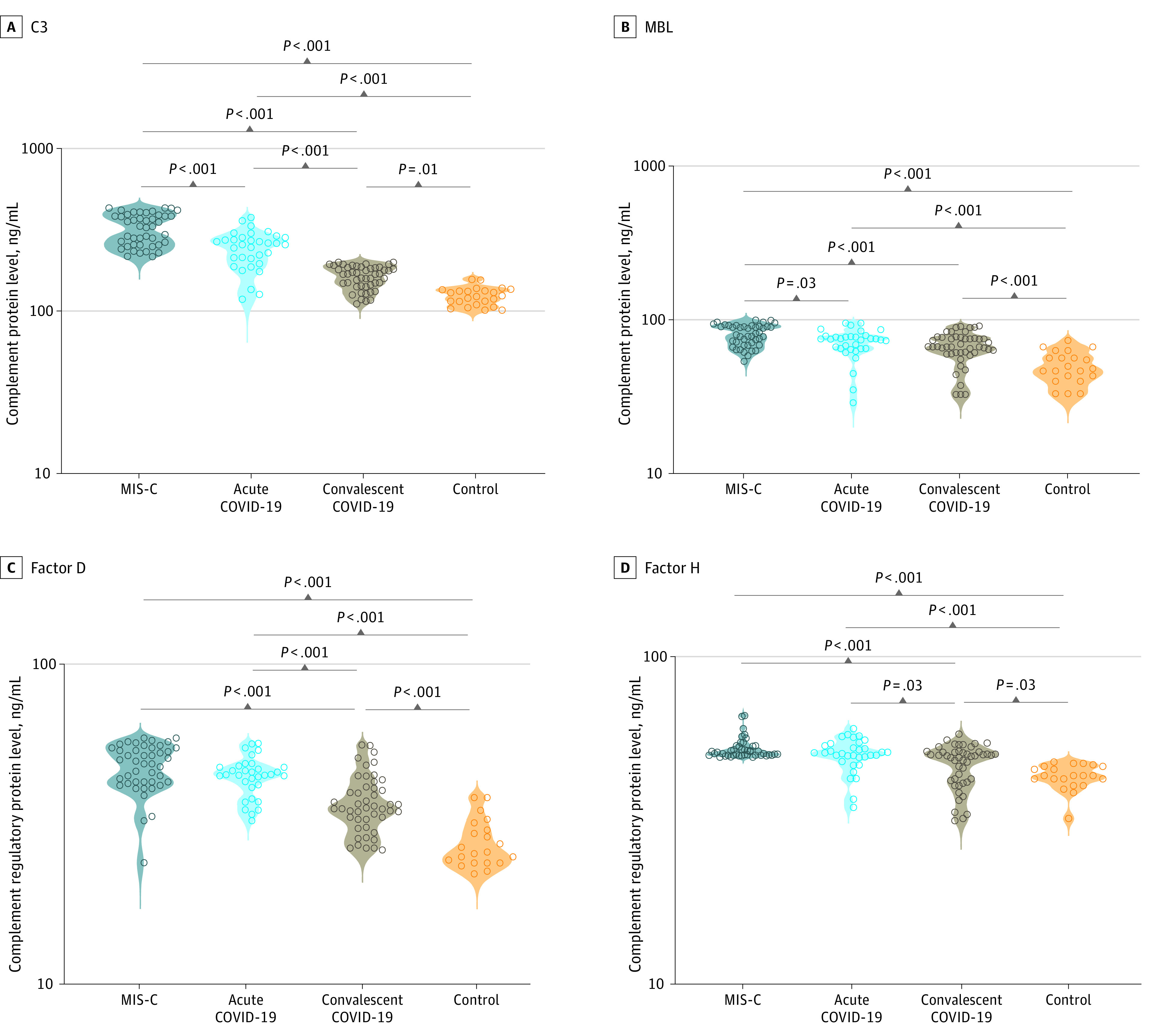
Complement Components and Regulators Activation in Children With Multisystem Inflammatory Syndrome in Children (MIS-C) and Acute COVID-19 Groups included plasma samples from 44 children with MIS-C, 33 with acute COVID-19, 47 with convalescent COVID-19, and 21 in the control group. Each circle represents a single individual. *P* values were calculated using the Kruskal-Wallis test with Dunn post hoc for multiple comparisons. MBL indicates mannose-binding lectin.

We measured the plasma levels of C1q, C2, C3, C3b/iC3b, C4, C4b, C5, C5a, and MBL complement proteins as well as complement regulatory proteins like factor B, factor D, factor H, and factor I in all groups of children. Children with MIS-C, vs those with acute COVID-19, had significantly elevated GM levels of C3 (318.2 [70.7] ng/mL vs 237.7 [61.8] ng/mL), C5a (2614.0 [336.2] ng/mL vs 1826.0 [541.0] ng/mL), and MBL (79.0 [12.4] ng/mL vs 69.6 [14.7] ng/mL) (Figure 1, Figure 2, eFigure 1 and eTable 2 in Supplement 1). In comparison with convalescent children with COVID-19 and in the control group, children with MIS-C had significantly elevated GM levels of C1q (61.5 [18.5] ng/mL vs acute COVID-19, 56.9 [18.6] ng/mL; controls, 24.1 [3.3] ng/mL), C2 (605.8 [219.7] ng/mL vs acute COVID-19, 606.4 [167.7] ng/mL; controls, 255.9 [73.3] ng/mL), C4 (537.4 [87.3] ng/mL vs acute COVID-19, 539.1 [144.4] ng/mL; controls, 396.9 [169.3] ng/mL), MBL (79.4 [12.4] ng/mL vs acute COVID-19, 69.6 [14.7] ng/mL; controls, 49.3 [11.8] ng/mL), C3 (318.2 [70.7] ng/mL vs acute COVID-19, 237.7 [61.8] ng/mL; controls, 123.4 [15.7] ng/mL), C5 (1487.0 [302.5] ng/mL vs acute COVID-19, 1364.0 [253.6] ng/mL; controls, 561.9 [50.2] ng/mL), C3b/iC3b (3971.0 [635.1] ng/mL vs acute COVID-19, 3702.0 [653.9] ng/mL; controls, 2039.0 [344.5] ng/mL), C5a (2614.0 [336.2] ng/mL vs acute COVID-19, 1826.0 [541.0] ng/mL; controls, 462.5 [132.4] ng/mL), C4b (712.4 [163.3] ng/mL vs acute COVID-19, 640.7 [180.1] ng/mL; controls, 351.5 [80.0] ng/mL), factor B (47.6 [7.8] ng/mL vs acute COVID-19, 44.6 [6.3] ng/mL; controls, 27.5 [5.0] ng/mL), factor D (44.0 [17.2] ng/mL vs acute COVID-19, 33.8 [18.4] ng/mL; controls, 21.3 [6.1] ng/mL), factor H (53.1 [4.0] ng/mL vs acute COVID-19, 50.8 [5.7] ng/mL; controls, 43.6 [3.8] ng/mL), and factor I (242.4 [156.4] ng/mL vs acute COVID-19, 332.7 [135.9] ng/mL; controls, 233.9 [85.2] ng/mL). Thus, MIS-C exhibited elevated levels of certain complement components compared with the other 3 groups. Children with acute COVID-19 had significantly elevated levels of C1q, C2, C4, C3, C5, C5a, C3b, factor B, factor H, and factor I in comparison with convalescent COVID-19 children. Children with acute COVID-19 also had significantly elevated levels of C1q, C2, C4, C3, C5, C3b/iC3b, C4b, C5a, and MBL complement proteins and of complement regulatory proteins like factor B, factor D, factor H, and factor I in comparison with control children (eTable 2 in Supplement 1). Thus, children with acute COVID-19 exhibited elevated levels of certain complement components compared with convalescent and control children. Children with convalescent COVID-19 had significantly elevated levels of C1q, C2, C3, C3b/iC3b, C4b, C5, C5a, and MBL complement proteins and of complement regulatory proteins like factor B and factor H in comparison with children in the control group. Thus, children with MIS-C and acute COVID-19 exhibited markedly elevated levels of most complement components and regulators when compared with children who were convalescent or part of the control group.

### Association of Disease Severity With Complement Activation Among Children With MIS-C

**Figure 3.**
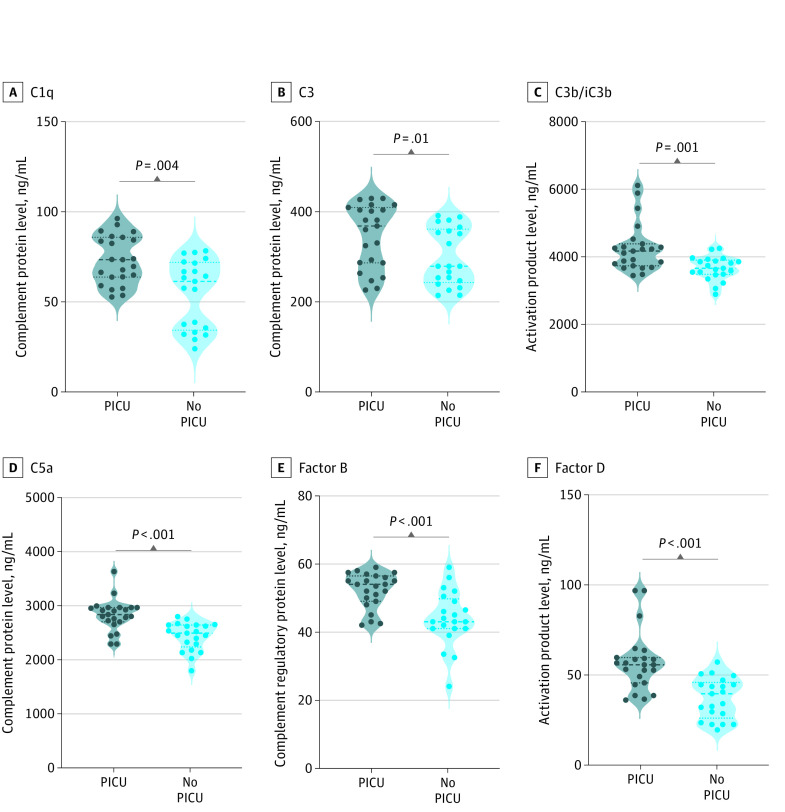
Enhanced Complement Activation and Disease Severity in Children With Multisystem Inflammatory Syndrome in Children (MIS-C) Groups included plasma samples from 23 children with MIS-C requiring pediatric intensive care unit (PICU) care, and 21 children in wards who did not require PICU care. Each circle represents a single individual. *P* values were calculated using the Mann- Whitney test with Holm correction for multiple comparisons.

Next, we wanted to determine the association of complement activation with disease severity in MIS-C. Therefore, we compared the admission (pretreatment) plasma levels of C1q (PICU, 72.3 [13.3] ng/mL vs no PICU, 51.5 [18.8] ng/mL), C2 (PICU, 718.9 [201.7] ng/mL vs no PICU, 502.2 [182.0] ng/mL), C4 (PICU, 585.7 [75.1] ng/mL vs no PICU, 489.0 [71.6] ng/mL), C4b (PICU, 788.6 [151.6] ng/mL vs no PICU, 637.4 [140.4] ng/mL), MBL (PICU, 88.3 [7.5] ng/mL vs 70.8 [10.4] ng/mL), C5 (PICU, 1659.0 [265.8] ng/mL vs no PICU, 1320.0 [235.3] ng/mL), C5a (PICU, 2806.0 [290.1] ng/mL vs no PICU, 2418.0 [260.2] ng/mL), C3 (PICU, 341.1 [70.7] ng/mL vs no PICU, 294.9 [63.4] ng/mL), C3b (PICU, 4247.0 [710.9] ng/mL vs no PICU, 3690.0 [345.1] ng/mL), factor B (PICU, 52.0 [5.2] ng/mL vs no PICU, 43.2 [8.0] ng/mL), factor D (PICU, 54.4 [16.3] ng/mL vs no PICU, 34.8 [11.3] ng/mL), factor H (PICU, 54.6 [4.9] ng/mL vs no PICU, 51.5 [0.9] ng/mL), and factor I (PICU, 300.2 [180.7] ng/mL vs no PICU, 191.7 [76.7] ng/mL) in children with MIS-C requiring PICU care (indicating severe disease) and children who did not need PICU care (indicating mild or moderate disease). The complement proteins C1q, C2, C4, C4b, MBL, C5, C5a, C3, and C3b and complement factors such as factor B, factor D, factor H, and factor I were significantly enhanced in children with MIS-C requiring PICU care compared with those who did not (Figure 3; eFigure 2 and eTable 3 in Supplement 1). Thus, these results indicate that these analyte levels exhibit a significant association with disease severity in MIS-C.

### Association of Disease Severity With Enhanced Complement Activation Among Children With Acute COVID-19

**Figure 4.**
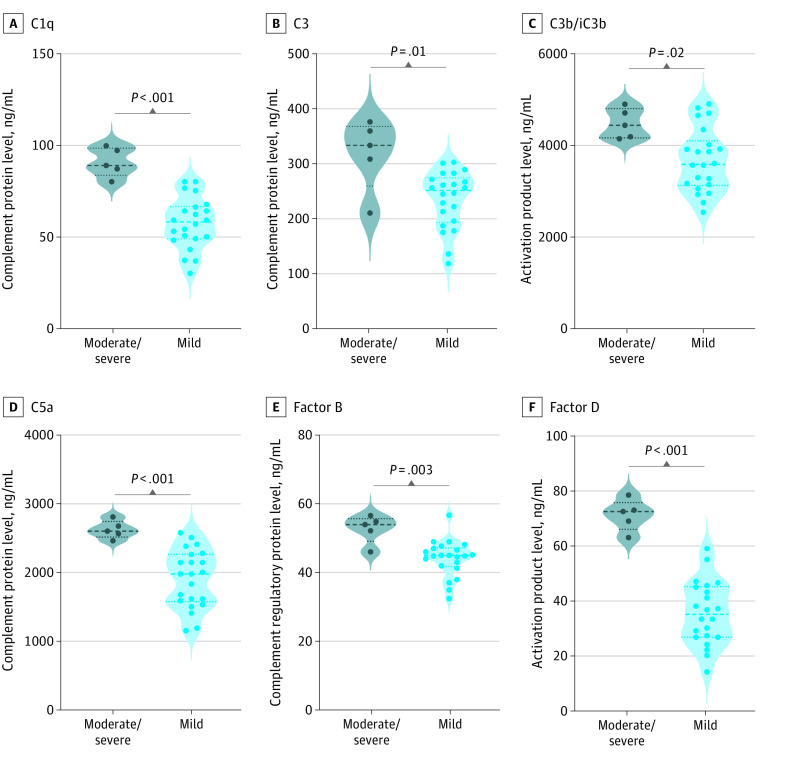
Enhanced Complement Activation and Disease Severity in Children With Acute COVID-19 Groups included plasma samples for 5 children with moderate to severe COVID-19, 22 with mild COVID-19, and 5 with asymptomatic COVID-19. Each circle represents a single individual. *P* values were calculated using the Kruskal-Wallis test with Dunn post hoc for multiple comparisons.

We next sought to ascertain if complement activation was associated with disease severity in acute COVID-19. Therefore, we compared the admission (pretreatment) plasma levels of C1q, C2, C4, C4b, MBL, C5, C5a, C3, C3b, factor B, factor D, factor H, and factor I between children with COVID-19 who developed moderate or severe disease and those who developed only mild disease. The complement proteins C1q, C2, C4, C4b, MBL, C5, C5a, C3, C3b and complement factors such as factor B, factor D, factor H, and factor I were significantly enhanced in children with severe or moderate COVID-19 (Figure 4; eFigure 3, eTable 5, eTable 6 in Supplement 1). Thus, these results indicate that these analyte levels exhibited a significant association with disease severity in MIS-C.

### Association of Plasma Complement Components, Complement Regulators, and Complement Activation Products With Laboratory and Biochemical Parameters and Pro-Inflammatory Cytokines

**Figure 5.**
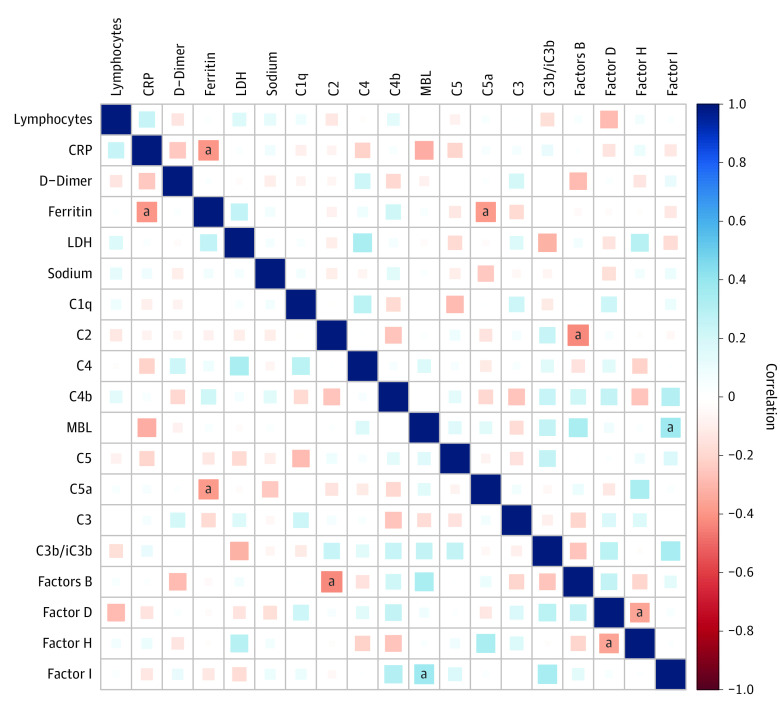
Correlation of Plasma Complement Components, Complement Regulators, and Complement Activation Products With Laboratory Parameters and Pro-Inflammatory Cytokines CRP indicates C-reactive protein; LDH, lactate dehydrogenase. ^a^Indicates *P* < .05. the blue color denotes the positive correlation and the red color denotes the negative correlation.

Next, we wanted to determine the association of plasma complement components, complement regulators, and complement activation products with biochemical and hematological parameters. Therefore, we performed the Spearman rank correlation analysis to determine the association of plasma immune markers with C-reactive protein, lymphocyte count, sodium, D-dimer, ferritin, and lactate dehydrogenase (Figure 5). Ferritin exhibited a negative correlation with C5a, and factor B exhibited a negative correlation with C2, whereas factor I exhibited a positive correlation with MBL. Furthermore, we wanted to examine the possible association between pro-inflammatory cytokines and the systemic levels of complement components, complement regulators, and complement activation products. The cytokine data which we used were previously published with the same cohort.^26^ Plasma levels of C1q, C2, C3, C4, C4b, C5, C5a, C3b/iC3b, and factors B, D, and H exhibited a positive correlation with the pro-inflammatory cytokines interferon (IFN) γ, interlukin (IL) 2, tumor necrosis factor α, IL-1α, IFNα, IFNβ, IL-6, IL-15, IL-17 and granulocyte macrophage colony-stimulating factor (eFigure 4 in Supplement 1). These results imply that complement components, complement regulators, and complement activation products exhibit a significant association with biochemical parameters and pro-inflammatory cytokines in MIS-C and acute COVID-19.

## Discussion

In this cross-sectional study, complement components, regulators, and activation products were significantly elevated in children with MIS-C and acute COVID-19. Earlier reports of previous coronaviral infections have revealed that viruses (SARS-CoV, MERS-CoV, and SARS-CoV-2) could bind mannose-binding protein-related serine protease 2 (MASP-2) and stimulate complement-mediated inflammatory lung damage.^31^ However, the inhibition of complement C3 could potentially decrease the inflammatory responses to pulmonary infections.^32,33^ The activation of the alternative pathway by the spike glycoprotein S of SARS-CoV-2 promotes entry of the virus into cells and is a major antigenic target for B cell responses.^34,35,36^ Previous adult studies^37^ have shown that complement activation has been associated with respiratory failure, acute respiratory distress syndrome development, and the severity of bacterial and viral pneumonia. The complement classical pathway can be triggered either directly by the pathogen or indirectly through antibodies bound to viral antigens, leading to the accumulation of C3b in the viral envelope.^38^

Porritt et al^15^ showed that children with MIS-C had elevated levels of classical complement pathway component C1q. Studies describing the plasma and serum proteomic analysis revealed that many complement components and their regulators were significantly upregulated in adult patients with COVID-19.^18,39,40,41,42^ Diorio et al^24^ observed that soluble C5b-9,^13,22^ MBL pathway, C3, and C4 levels were elevated in children with SARS-CoV-2 infection,^43^ and complement-mediated thrombotic microangiopathy (TMA) has been associated with SARS-CoV-2 infection.^23^ A 2022 study^44^ determined that the MIS-C group exhibited increased frequencies of the complement factor H coding single nucleotide variants potentially predisposing to complement deregulation. Concurrent with the existing studies, we observed elevated levels of complement pathways components (C1q, C2, and C4) in children with MIS-C and acute COVID-19. As C1q is driven by immune complex interactions, it is possible immune complex–mediated pathology, including from complement pathways, is enhanced in MIS-C and acute COVID-19.^15,45^ In children with MIS-C, the poorer opsonization of the virus could lead to slower and compromised immune clearance with persistent systemic inflammation associated with severe infection.^46^

It is known that the circulating viral particles can trigger complement activation through the MBL pathway and cause microvascular injury in the adult COVID-19.^47^ Magro et al^11^ found that mannose-binding protein-associated serine protease 2 (MASP-2) deposits were present in biopsies of lung and skin tissue, thus highlighting a potential contribution of the complement pathway to the process of thrombotic microvascular injury through endothelial damage and subsequent activation of the coagulation cascade. SARS-CoV-2 itself interacts with MBL to activate complement C3 proximally through the lectin pathway on virus-infected cells.^48^ Likewise, SARS-CoV-2 secreted nucleoprotein binds to MASP-2, the main component of the lectin pathway, which can exacerbate inflammatory lung injury.^49^ The nucleocapsid protein also aggravates lung injury through the MASP-2–mediated complement hyperactivation.^50^ Noris et al^43^ reviewed earlier reports that determined that proteins of SARS-CoV-2 have mannose groups recognized by the mannose-binding lectin complement pathway, and autopsy specimens of lungs from patients who have died of COVID-19 exhibited immunohistochemistry staining for mannose-binding lectin, C3, C4, and soluble C5b9. Similar to the adult findings, we found that children with MIS-C and acute COVID-19 had high levels of MBL, suggesting the involvement of complement components in pediatric COVID-19 and MIS-C.

The main function of the alternative pathway is to amplify the initial activation from the classical and lectin pathway through the central C3 component, which, in turn, activates C5. Activation of C5 then leads to the formation of the potent anaphylatoxin C5a and the terminal C5b-9 complement complex, both exerting proinflammatory actions such as recruitment of neutrophils, activation of the adaptive immune system, and endothelial cell activation.^51,52^ The terminal complement components C5a could stimulate endothelial injury and dysfunction through multiple processes.^53^ It has been previously found that the levels C3 and C5 levels were elevated in severe COVID-19 adult patients.^54^ Diorio et al^24^ observed that soluble C5b-9,^13,22^ complement activation and complement-mediated thrombotic microangiopathy (TMA) have been associated with SARS-CoV-2 infection.^23^ In our cohort of children, we found that the levels of C3 and C5a levels were elevated in children with MIS-C and acute COVID-19, which suggests that prolonged and excessive activation in the host may be what leads to pathology.

Azafi et al^53^ showed that elevated levels of complement activity play a key role in the pathogenesis of COVID-19. We observed a significant difference in the complement hyperactivation of children with MIS-C requiring PICU support in comparison with those who did not require PICU support. The complement components C1q, C3, complement regulator factor D, factor I, and activation product C3b/iC3b exhibited associations with lymphocytes, C-reactive protein, and sodium levels, respectively, which have been described as markers associated with the prognosis of MIS-C and acute COVID-19.^13^ These results highlight the possible association of the complement system with the disease severity of MIS-C and COVID-19 in children. The association between the complement system and proinflammatory cytokines is mutual and feed-forward. Various studies implied that proinflammatory cytokines increase the expression of anaphylatoxin receptors in inflammatory cells.^55^ The consequence of anaphylatoxins on cytokine expression is dependent severely on the pathophysiological perspective of the enduring inflammatory response.^51,55^ Our results suggest that the hyperactivation of the complement system and heightened levels of proinflammatory cytokines significantly correlated with the severity of MIS-C.

### Limitations

Our study had several limitations. We included children from a single institution with a small number of children in all groups, and we were unable to assess the immunological profile with longitudinal follow-up. Our study was also descriptive in nature and did not delineate underlying mechanisms.

## Conclusions

Our data indicated that complement hyperactivation may play a vital role in the pathogenesis of MIS-C and acute COVID-19. Our data also indicated that complement levels exhibit possible associations with disease severity in MIS-C and COVID-19 and might be potential biomarkers of pathogenesis. As complement activation could promote immune-mediated organ damage, further studies are warranted to establish whether complement inhibition might have possible therapeutic advantages in a hyperinflammatory phenotype, especially in children with MIS-C.

## Notes

Supplement 1. **eFigure 1.** MIS-C and Acute COVID-19 Children Are Associated With Elevated Levels of Complement Activation **eFigure 2.** Enhanced Complement Activation Is Associated With Disease Severity in MIS-C Children **eFigure 3.** Enhanced Complement Activation Is Associated With Disease Severity in Acute COVID-19 **eFigure 4.** Correlation of Plasma Complement Components, Complement Regulators and Complement Activation Products With Laboratory Parameters and Pro-inflammatory Cytokines **eTable 1.** Demographics and Haematology Parameters of the Study Population **eTable 2.** Geo Mean and Fold Change of Complement Components of the Study Groups **eTable 3.** Geo Mean and Fold Change of Complement Components Between PICU vs No PICU **eTable 4.** Geo Mean and Fold Change of Complement Components Between Moderate/Severe Vs Mild **eTable 5.** Geo Mean and Standard Deviation of Complement Activation Is Associated With Disease Severity in Acute COVID-19 **eTable 6.** Geo Mean and Standard Deviation of Complement Activation Is Associated With Disease Severity in Acute COVID-19

Supplement 2.
Data Sharing Statement
