# Supplementary material for: Levels of Complement Components in Children With Acute COVID-19 or Multisystem Inflammatory Syndrome
Source: JAMA Netw Open. 2023 Mar 24;6(3):e231713. doi: 10.1001/jamanetworkopen.2023.1713 (PMC10313152; doi:10.1001/jamanetworkopen.2023.1713)
Supplement: Supplement 1. — eFigure 1. MIS-C and Acute COVID-19 Children Are Associated With Elevated Levels of Complement Activation eFigure 2. Enhanced Complement Activation Is Associated With Disease Severity in MIS-C Children eFigure 3. Enhanced Complement Activation Is Associated With Disease Severity in Acute COVID-19 eFigure 4. Correlation of Plasma Complement Components, Complement Regulators and Complement Activation Products With Laboratory Parameters and Pro-inflammatory Cytokines eTable 1. Demographics and Haematology Parameters of the Study Population eTable 2. Geo Mean and Fold Change of Complement Components of the Study Groups eTable 3. Geo Mean and Fold Change of Complement Components Between PICU vs No PICU eTable 4. Geo Mean and Fold Change of Complement Components Between Moderate/Severe Vs Mild eTable 5. Geo Mean and Standard Deviation of Complement Activation Is Associated With Disease Severity in Acute COVID-19 eTable 6. Geo Mean and Standard Deviation of Complement Activation Is Associated With Disease Severity in Acute COVID-19 [file jamanetwopen-e231713-s001.pdf]

Supplemental Online Content

Rajamanickam A, Nathella PK, Venkataraman A, et al. Levels of complement components in children with acute COVID-19 or multi-system inflammatory syndrome. *JAMA Netw Open.* 2023;6(3):e231713. doi:10.1001/jamanetworkopen.2023.1713

eFigure 1. MIS-C and Acute COVID-19 Children Are Associated With Elevated Levels of Complement Activation

eFigure 2. Enhanced Complement Activation Is Associated With Disease Severity in MIS-C Children

eFigure 3. Enhanced Complement Activation Is Associated With Disease Severity in Acute COVID-19

eFigure 4. Correlation of Plasma Complement Components, Complement Regulators and Complement Activation Products With Laboratory Parameters and Pro-inflammatory Cytokines

eTable 1. Demographics and Haematology Parameters of the Study Population

eTable 2. Geo Mean and Fold Change of Complement Components of the Study Groups

eTable 3. Geo Mean and Fold Change of Complement Components Between PICU vs No PICU

eTable 4. Geo Mean and Fold Change of Complement Components Between Moderate/Severe Vs Mild

eTable 5. Geo Mean and Standard Deviation of Complement Activation Is Associated With Disease Severity in Acute COVID-19

eTable 6. Geo Mean and Standard Deviation of Complement Activation Is Associated With Disease Severity in Acute COVID-19

This supplemental material has been provided by the authors to give readers additional information about their work.

© 2023 Rajamanickam A et al. *JAMA Network Open.*

eFigure 1. MIS-C and acute COVID-19 children are associated with elevated levels of complement activation

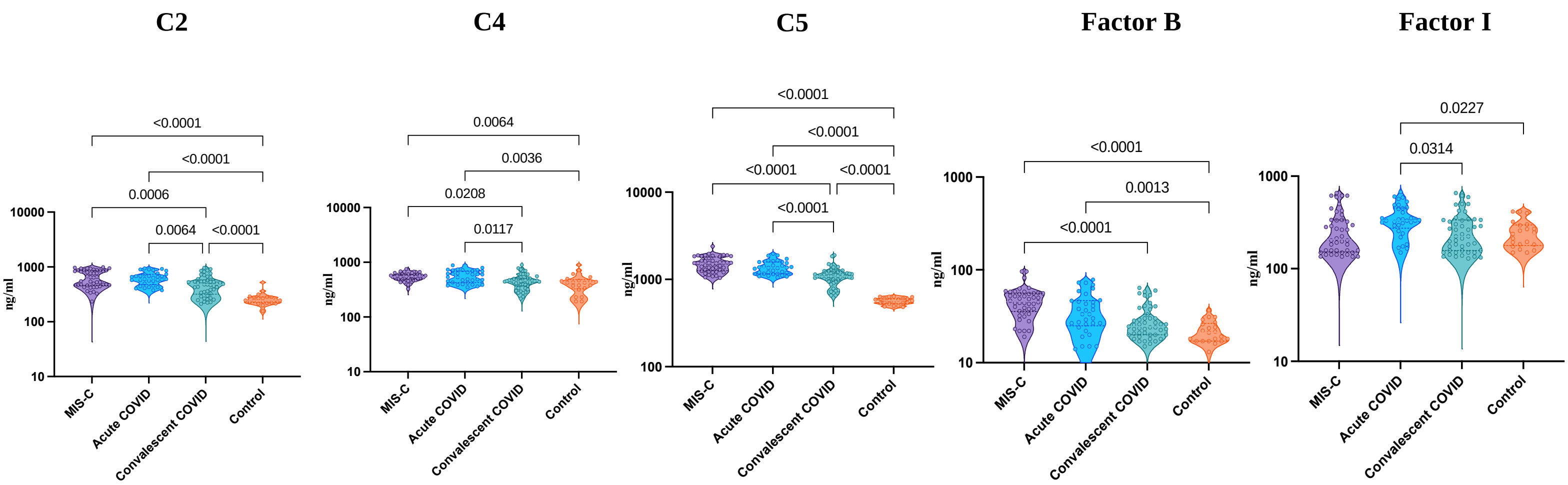

eFigure.1. Levels of the specified complement components, complement regulators and complement activation products C2, C4, C4b and MBL, C5, and iC3b, factor B, factor D, and factor I in plasma samples from MIS-C (n=44), Acute COVID-19 (n=33), convalescent COVID-19 (n=47) and control (n=21) children. The data are represented as scatter plots with each circle representing a single individual. P values were calculated using the Kruskal-Wallis test with Dunn's post-hoc for multiple comparisons.

© 2023 Rajamanickam A et al. *JAMA Network Open.*

eFigure 2. Enhanced complement activation is associated with disease severity in MIS-C children

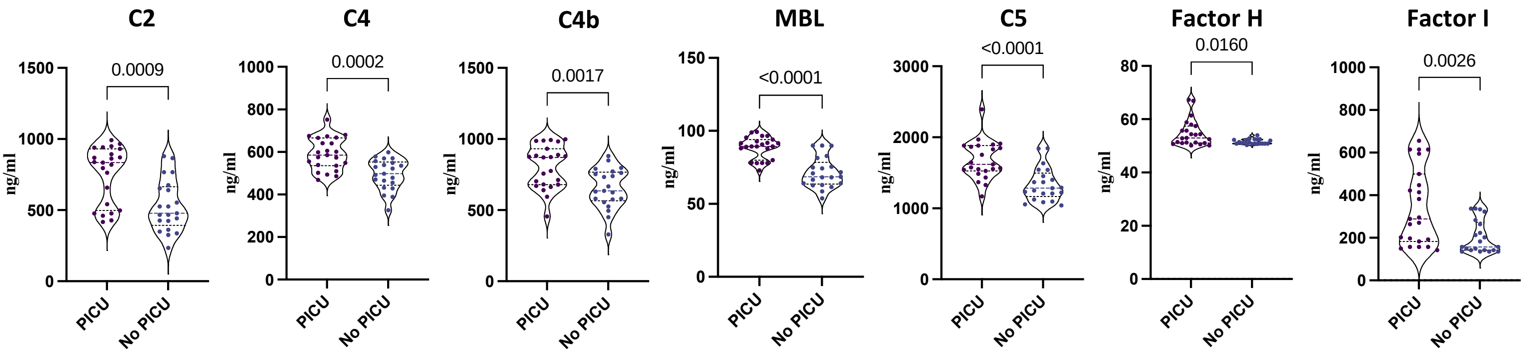

eFigure.2. Levels of the specified complement components, complement regulators and complement activation products C2, C4, C4b, MBL, C5, factor H and factor I were measured using plasma samples in children with MIS-C requiring PICU care (n = 23) and children in wards [who did not require PICU care] (n = 21). The data are represented as scatter plots with each circle representing a single individual. p values were calculated using the Mann- Whitney test with Holm's correction for multiple comparisons.

© 2023 Rajamanickam A et al. *JAMA Network Open.*

eFigure 3. Enhanced complement activation is associated with disease severity in acute COVID-19

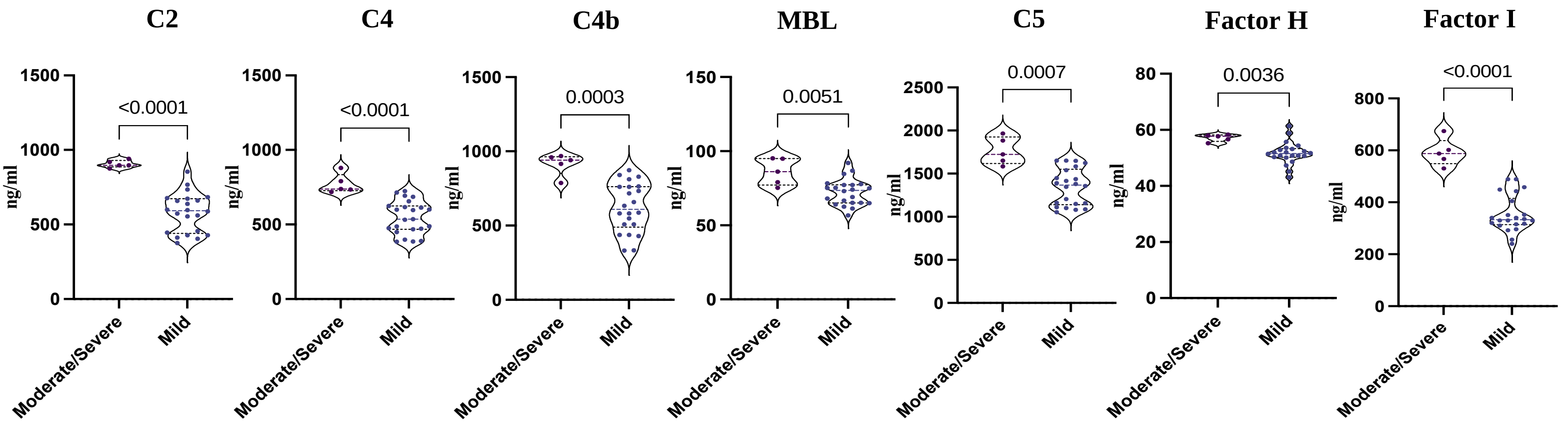

eFigure.3. Levels of the specified complement components, complement regulators and complement activation products C2, C4, C4b, MBL, C5, factor H and factor I were measured using plasma samples in children with moderate to severe (n = 5) COVID-19, and children with mild (n = 22) and asymptomatic (n = 5) COVID-19. The data are represented as scatter plots with each circle representing a single individual. P values were calculated using the Kruskal—Wallis test with Dunn's post—hoc for multiple comparisons.

© 2023 Rajamanickam A et al. *JAMA Network Open.*

eFigure 4. Correlation of plasma complement components, complement regulators and complement activation products with laboratory parameters and pro-inflammatory cytokines

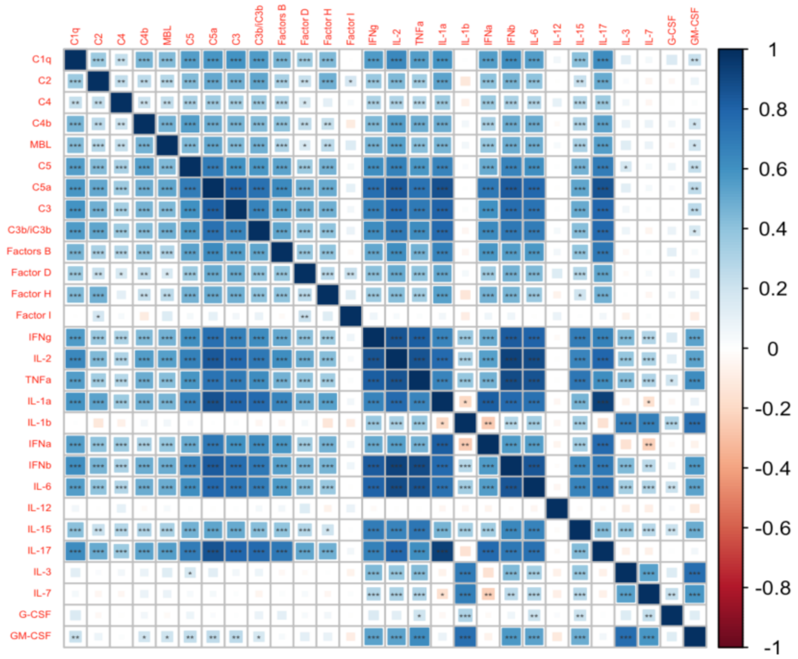

eFigure.4. Correlation between plasma levels of complement components (C1q, C2, C3, C4, C4b, MBL, C5), regulators (factor B, factor D, factor H and factor I) and activation products (iC3b, C5b) and pro-inflammatory cytokines (IFNγ, IL-2, TNFα, IL1α, IL1β, IFNα, IFNβ, IL-6, IL-12, IL-15, IL-17, IL-3, IL-7, G-CSF and GM-CSF) levels in all children with MIS-C, acute COVID-19, convalescent COVID-19 and control. Spearman's correlation method was used for the analysis. * indicates p=0.05, ** indicates p= 0.01 and *** indicates p=0.001 the blue colour denotes the positive correlation and the red colour denotes the negative correlation.

© 2023 Rajamanickam A et al. *JAMA Network Open.*

eTable 1. Demographics and haematology parameters of the study population

	MIS-C n = 44	Acute COVID-19 n = 29	Convalescent COVID-19 n = 47	Controls n=21
**Age median (years, IQR)**	7 y (1 – 14y)	5 (1 – 17 yr)	4.4 y (1 – 17 y)	6 y, (1 – 15 y
**Male n (%)**	19 (43%)	20 (60%)	35 (74%)	11 (52%)
**Female n (%)**	25 (57%)	9 (40%)	12 (26%)	10 (48%)
**Serology IgG positive n (%)**	44 (100%)	0	47 (100%)	0
CRP mean (< 5 mg/L)	169 (39 – 473)	5 (<5 – 181)	5 (<5 – 181)	NA
Lymphocyte(/mm3) (1500 – 4000) median (IQR)	1386 (330 - 2200)	2945 (650 – 12000)	3890 (650 – 12000)	3200 (2600 – 4000)
Neutrophils (/mm3) (1500 – 7000) median (IQR)	11658 (9918 - 14878)	3681 (120 – 13160)	6300 (120 – 13160)	4500 (3500 – 6500)
Platelets (200 – 450)x10^9/L median (IQR)	110 (62 – 210)	278 (180 – 400)	327 (100 – 540)	350 (350 -450)
Sodium (135 – 145mmol/l) median (IQR)	134 (127 -138)	136 (135 – 145)	138 (135 – 148)	139 (135 – 145)
D-Dimer(100–500ng/ml FEU) median (IQR)	4890 (2446–10 000)	NA	NA	NA
Ferritin (7 to 140ng/mL) median (IQR)	605 (38–2571)	NA	NA	NA
LDH (125–243U/L) median (IQR)	494 (200–905)	NA	NA	NA

© 2023 Rajamanickam A et al. *JAMA Network Open.*

eTable 2. Geo Mean and Fold change of complement components of the study groups

Parameters	MIS-C Geo Mean ng/ml	Acute COVID-19 Geo Mean ng/ml	Convalescent COVID-19 Geo Mean ng/ml	Control Geo Mean ng/ml	Acute COVID-19 Fold change	Convalescent COVID-19 Fold change	Control Fold change
**C1q**	61.48	56.92	41.57	24.14	1.1	1.5	2.5
**C2**	605.8	606.4	448.1	255.9	1.0	1.4	2.4
**C4**	537.4	539.1	441.8	396.9	1.0	1.2	1.4
**C4b**	712.4	640.7	562.3	351.5	1.1	1.3	2.0
**MBL**	79.42	69.56	65.02	49.32	1.1	1.2	1.6
**C5**	1487	1364	1076	561.9	1.1	1.4	2.6
**C5a**	2614	1826	886	462.5	1.4	3.0	5.7
**C3**	318.2	237.7	161.1	123.4	1.3	2.0	2.6
**C3b/iC3b**	3971	3702	2646	2039	1.1	1.5	1.9
**Factor B**	47.62	44.58	36.64	27.53	1.1	1.3	1.7
**Factor D**	43.95	33.84	26.89	21.28	1.3	1.6	2.1
**Factor H**	53.11	50.82	47.23	43.62	1.0	1.1	1.2
**Factor I**	242.4	332.7	242	233.9	0.7	1.0	1.0

© 2023 Rajamanickam A et al. *JAMA Network Open.*

eTable 3. Geo Mean and Fold Change of Complement Components Between PICU vs No PICU

Parameters	PICU<br>Geo Mean ng/ml	No PICU<br>Geo Mean ng/ml	Fold change
**C1q**	72.31	51.47	1.4
**C2**	718.9	502.2	1.4
**C4**	585.7	489	1.2
**C4b**	788.6	637.4	1.2
**MBL**	88.26	70.75	1.2
**C5**	1659	1320	1.3
**C5a**	2806	2418	1.2
**C3**	341.1	294.9	1.2
**C3b/iC3b**	4247	3690	1.2
**Factor B**	52.02	43.22	1.2
**Factor D**	54.44	34.77	1.6
**Factor H**	54.58	51.54	1.1
**Factor I**	300.2	191.7	1.6

© 2023 Rajamanickam A et al. *JAMA Network Open.*

eTable 4. Geo Mean and Fold change of complement components between Moderate/Severe Vs Mild

Fig.3	Moderate/Severe	Mild	Fold change
**C1q**	89.44	54.71	1.6
**C2**	905.8	565.8	1.6
**C4**	770	536.4	1.4
**C4b**	911.5	597.9	1.5
**MBL**	85.75	71.78	1.2
**C5**	1756	1329	1.3
**C5a**	2608	1845	1.4
**C3**	311.8	229.3	1.4
**C3b/iC3b**	4450	3597	1.2
**Factor B**	52.71	44	1.2
**Factor D**	71.01	33.55	2.1
**Factor H**	57.16	51.54	1.1
**Factor I**	590.5	347.7	1.7

© 2023 Rajamanickam A et al. *JAMA Network Open.*

eTable 5. Geo Mean and Standard deviation of complement activation is associated with disease severity in acute COVID-19.

eFig.3	Moderate/Severe GM and SD	Mild	p value
**C2**	905.8 [131.0]	565.8 [24.6]	<0.0001
**C4**	770 [110.5]	536.4 [65.8]	<0.0001
**C4b**	911.5 [166.3]	597.9 [74.0]	0.0003
**MBL**	85.8 [8.8]	71.8 [9.0]	0.0051
**C5**	1756.0 [159.4]	1329.0 [212.1]	0.0007
**Factor H**	57.2 [4.0]	51.5 [1.3]	0.0036
**Factor I**	590.5 [70.7]	347.7 [53.0]	<0.0001

© 2023 Rajamanickam A et al. *JAMA Network Open.*

eTable.6. Geo Mean and Standard deviation of complement activation is associated with disease severity in acute COVID-19.

Fig.4	Moderate/Severe GM and SD	Mild	p value
**C1q**	89.4 [14.0]	54.7 [8.0]	<0.0001
**C3**	311.8 [65.3]	229.3 [51.8]	0.0121
**C3b/iC3b**	4450.0 [692.5]	3597.0 [327.1]	0.0152
**C5a**	2608.0 [420.8]	1845.0 [128.8]	0.0002
**Factor B**	52.7 [5.3]	44.0 [4.0]	0.0034
**Factor D**	71.0 [11.5]	33.6 [5.7]	<0.0001

© 2023 Rajamanickam A et al. *JAMA Network Open.*
