# Supplementary material for: Levels of Complement Components in Children With Acute COVID-19 or Multisystem Inflammatory Syndrome
Source: JAMA Netw Open. 2023 Mar 24;6(3):e231713. doi: 10.1001/jamanetworkopen.2023.1713 (PMC10313152; doi:10.1001/jamanetworkopen.2023.1713)
Supplement: Supplement 2. — Data Sharing Statement [file jamanetwopen-e231713-s002.pdf]

Data Sharing Statement

Rajamanickam. Levels of Complement Components in Children With Acute COVID-19 or Multisystem Inflammatory Syndrome. *JAMA Netw Open*. Published March 06, 2023. doi:10.1001/jamanetworkopen.2023.1713

Data
Data available: No

Additional Information
Explanation for why data not available: All the reported data are available within the manuscript
